# Identification of Genes Involved in Wild Crucifer *Rorippa indica* Resistance Response on Mustard Aphid *Lipaphis erysimi* Challenge

**DOI:** 10.1371/journal.pone.0073632

**Published:** 2013-09-09

**Authors:** Lekha Bandopadhyay, Debabrata Basu, Samir Ranjan Sikdar

**Affiliations:** Division of Plant Biology, Bose Institute, Centenary Campus, Kolkata, India; Nanjing Agricultural University, China

## Abstract

Mustard aphid, *Lipaphis erysimi* (L.) Kaltenbach is a perpetual annual threat in the cultivation of rapeseed- mustard (*Brassica spp*.) crop in tropical and sub-tropical climate. Cultivated *Brassica* germplasm has failed so far to provide any source of resistance. Wild germplasm is a potential source of resistance against many threatening herbivores. On wild germplasm screening, we noted that the wild crucifer *Rorippa indica* (L.) Hiern confers resistance against *L. erysimi*. In the present study *L. erysimi* challenged transcriptome of *R. indica* was compared to un-infested *R. indica* sample to get a molecular insight about the aphid resistance mechanism and identify the candidate defense response genes. Cloning, sequencing and *in silico* sequence analysis of complimentary DNA amplified fragment length polymorphism identified 116 differentially expressed transcript derived fragments revealed thirty candidates which are from different functional categories including redox regulation, signalling, photosynthesis, structure, metabolism, defense response as well as a few of unknown function. Twenty four identifications were then studied by quantitative real time RT PCR analysis at 6, 12, 24 and 48 hour time point post infestation to understand the early-to-late defense response through their relative gene expression profiles. Seventeen fragments showed significant up or down regulation at p<0.05 level. The response was influenced by different phytohormonal signalling pathways simultaneously. The candidate defense response expressed sequence tags specifically for the resistance genes identified in this study have implication in building desired mustard aphid resistance in susceptible rapeseed-mustard plants in future. This is the first molecular report on crucifer defense response against mustard aphid *L. erysimi*.

## Introduction

Rapeseed-mustard (*Brassica spp*.) is the third most important oilseed crop after soybean (*Glycine max*) and palm (*Elaeis guineensis*) in world agriculture and India is the third largest producer with global contribution of 28.3% acreage and 19.8% production. Indian mustard, *Brassica juncea* (L.) Czern is the predominant cultivated variety in India with yield potential of 1500–3000 Kg/Hectare and 38–42% oil content. However, different biotic and abiotic factors affect consistency of rapeseed-mustard cultivation in India creating a difference between production potential and actual production [1].

Mustard aphid, *Lipaphis erysimi* (L.) Kaltenbach (Homoptera: Aphididae) is one of the most devastating pests of *B. juncea*. These tiny intruders are mostly visible at the flowering stage affecting the crop yield severely every year in terms of both quality and quantity [1], [2]. They are crucifer specialist like the more studied one *Brevicoryne brassicae* (cabbage aphid), but work best in the tropical climate on the oilseed *Brassicas*. Most alarmingly they serve as the vector of disease causing viruses of the family Luteoviridae [3]. The much discussed aphid repellent crucifer-glucosinolates have the least effect on these specialists. Instead they sequester plant glucosinolates and synthesise their own myrosinases for their own defense [4].

Cultivated *Brassica* germplasm has failed to provide any source of resistance against *L. erysimi*. Farmers maintain a standard harvest by frequent application of hazardous chemical pesticides. This unsustainable practice is, however, associated with the ubiquitous risk of pest resurgence, outbreak of secondary pest and eventual emergence of pesticide resistance [5]. Homopteran aphid performance is not affected by *Bacillus thuringiensis* encoded insecticidal crystal proteins (Bt-toxins) effective against Lepidopteran insects [6]. The insecticidal mannose-binding lectins from garlic (*Allium sativum* L. leaf agglutinin, ASAL) [7], [8] and onion (*Allium cepa* L. agglutinin, ACA) [9] along with the chitin-binding lectin from wheat (wheat germ agglutinin, WGA) [10] have been noted to be effective against homopteran sap sucking insects including *L. erysimi*. However, the results are yet to reach the field. Wild *Brassica*s like *Brassica fruticulosa* and *Brassica Montana* with high lectin content have been reported to show resistance against mustard aphid *L. erysimi* [11].

The wild relatives of cultivated crops often show resistance to several potential pests and pathogens. Resistance genes from wild have been reported to provide resistance against phylogenetically distinct pests and pathogens. Notably, NBS-LRR class of *Mi1.2* gene [identified from wild tomato *Lycopersicon peruvianum* (L.) P. Mill] is one such cloned insect resistance gene that confers resistance to potato aphid (*Macrosiphum euphorbiae*), root-knot nematodes (*Meloidogyne spp*.) [12] and whitefly (*Bemisia tabaci*) [13]. On wild germplasm screening, we noted that *Rorippa indica* (L.) Hiern, an ocassional shade loving weed shows resistance against the mustard aphid. *R. indica* is a wild crucifer found in the Indian subcontinent and Asia. It remains in the rosette form throughout the winter. Then it bolts out and grows into highly branched bush throughout the summer. It survives many non-specialist herbivores including the crucifer specialist aphid *L. erysimi* [14]. *R. indica* has been reported to contain a number of potential allelochemicals. These include sulphur and nitrogen containing compounds like hirsutin, arabin, camelin, roripamine (sulfonylalkylamine) [15] and three novel ω-methylsulfonylalkyl isothiocyanates (*n* = 8, 9, and 10) [16]. Flower nectar of *R. indica* is an attractant for some ant species. These ants feed on herbivorous insects on the plant. The attracted ants thus provide indirect defense against herbivorous insects like *Pieris* butterfly larvae. The study points a mutualistic relationship between *R. indica* and ants [17]. A report also indicated intra-guild predation interaction (IGP) between ants and herbivorous insects like larvae of the diamondback moth, *Plutella xylostella* on *R. indica* [18]. The reproductive plasticity of *R. indica* has been noted as their other wild survival strategy. In response to heavy leaf damage they allocate more resource to seed production at the cost of roots. In this way, they can escape unfavourable habitats by means of seed dispersal and seed dormancy [19]. In order to transfer the resistance trait from *R. indica* to *Brassica juncea*, we carried out somatic hybridization between these sexually incompatible pair. The somatic hybrid and their *Brassica* type backcrossed progenies (backcrossed with *B. juncea*) also showed *R. indica* type resistance [20], [21].

In the present study we aim to identify the candidate defense response gene(s) from *L. erysimi* resistant wild crucifer *R. indica*. Thus we have explored the early-to-late defense response in the *R. indica-L. erysimi* incompatible interaction by cDNA amplified fragment length polymorphism (cDNA AFLP) analysis. The identified differentially expressed transcript derived fragments (TDFs) were then subjected to a detailed time course relative gene expression level analysis by real time reverse transcriptase (RT) PCR. Besides gaining molecular insight about the resistant response in *R. indica*, the transcriptomic study offers some promising identifications in the context of building *L. erysimi* resistance in the consistently susceptible *Brassica*s. Most research on crucifer-aphid interaction till now has involved the crucifer feeding specialist *Brevicoryne brassicae* or the generalist *Myzus persicae* and another wild crucifer *Arabidopsis* [22]. This is the first molecular report on the crucifer defense response against the specialist mustard aphid *L. erysimi.*

## Materials and Methods

### Plant and Insect Materials

Surface sterilized seeds of *R. indica* were grown in sterile inorganic soil, Soilrite (KEL, India). Nutrient solution (Half strength Murashige & Skoog liquid medium without sucrose and organic components) [23] and sterile H_2_O were applied alternatively twice a week. The plants were grown at 25°C±2°C and 16/8 hour light/dark photoperiod for 60 days. Fresh *L. erysimi* colonies collected from infested mustard plants grown in the Institutional experimental farm were used for infestation in *R. indica* plant.

### Time Course Study of Aphid Infestation

Thirty aphids consisting of wingless adults and nymphs were gently placed on each plant with the aid of a soft paint brush. The extent of the infestation was studied after the aphids have settled on each plant (2 hours later) in terms of the resultant number of wingless aphids on each plant. Data was collected at 6, 12, 24, 36, 48 and 72 hours post infestation (hpi). The time course study was conducted with five plants for each time point. One way ANOVA followed by Tukey’s multiple comparison test at significance level of p<0.05 was carried out to determine the significance of the noted extent of infestation over the time course.

### Aphid Infestation and Sample Collection

The forced infestation process was carried out as described for the time course study. The whole aerial part of the aphid infested plants were harvested at different time intervals 12, 24, 48 and 72 hpi, snap-frozen in liquid nitrogen (−196°C) and stored at −80°C. Un-infested control plants were harvested and stored similarly.

### RNA Extraction and cDNA Synthesis

Total RNA was isolated from frozen tissue using TRIZOL Reagent (Invitrogen, US) separately for the 12, 24, 48 and 72 hpi time point collection. The quality of the isolated RNAs was checked by 1.4% agarose/EtBr gel electrophoresis. The quality was verified based on OD_260_/OD_280_ values and concentration was measured based on OD_260_ values using a ND-1000 Spectrophotometer (Nanodrop, Wilmington, DE, US). PolyA mRNA was purified with oligodT latex beads using NucleoTrap mRNA Mini Kit (MN, Germany) from pooled total RNA by mixing equal quantity of total RNA for each of the four time intervals. Double stranded cDNA was synthesized from 1 µg polyA mRNA using Super SMART PCR cDNA Synthesis Kit (Clontech, US). The quality was checked by 1.2% agarose/EtBr gel electrophoresis. The quality was verified based on OD_260_/OD_280_ values and concentration was measured based on OD_260_ values.

### cDNA AFLP Analysis

#### Reactions

500 ng of double stranded cDNA was used for cDNA AFLP analysis carried out with AFLP Analysis System I (Invitrogen, US). The analysis was done with two biological replicates each having two technical replicates. Selective amplification was carried out with 40 different combinations of EcoRI and MseI primers with three selective nucleotides (Table S1 & S2). The EcoRI selective primers were radiolabelled at the 5′ end by phosphorylating with [γ^−32^P] ATP, 1.41×10^14^ Bq/mmol (Jonaki BRIT, India) and T4 kinase for radioactive detection. All the amplifications were done on an Applied Biosystems 2720 Thermal cycler (US).

#### Gel analysis

[γ^−32^P] ATP labelled selective amplification products were separated on 6% denaturing polyacrylamide gels (20∶1 acrylamide : bis; 7.5 M urea; 1X TBE buffer) cast with 0.4 mm spacers and sharks tooth comb in a Sequi-Gen GT 21X40 cm gel apparatus (Biorad Laboratories). The gel was pre-electrophoresed for 45 minutes at constant power (50 W) and then loaded with 2 µl of the reaction product mixed with 2 µl loading buffer [98% formamide, 10 mM EDTA (pH 8.0), 0.025% (w/v) bromophenol blue, 0.025% (w/v) xylene cyanol]. 30–330 bp AFLP DNA Ladder (Invitrogen) was end labelled with [γ^−32^P] ATP with T4 polynucleotide kinase by exchange reaction and 2 µl labelled ladder was loaded along with the samples in the 6% polyacrylamide gel. Gels were dried on 3 MM CHR Paper (Himedia Laboratories, India) at 80°C on a slab gel drier (Genei, India). The autoradiograph of the cDNA AFLP profile was developed after exposing the dry gel to X-ray film (KODAK, XBT) at −80°C for 24 hours.

### Gel Elution, Extraction and Reamplification of cDNA AFLP Fragments

Selected differentially expressed cDNA AFLP fragments were carefully cut with a sterile scalpel blade from the dried polyacrylamide gels by superimposing with the respective autoradiograph-films. The elution was carried out as described in Frost and Guggenheim (1999) [24]. The eluted product was purified by phenol-chloroform extraction followed by isopropanol precipitation and 75% ethanol wash. To build sufficient template for downstream analysis, reamplification was performed with the eluted product with the respective EcoRI and MseI selective primers under the same PCR conditions with additional 15 min at 72°C for sufficient A-tailing by Taq DNA polymerase (without the 3′-5′ exonuclease activity), required in the subsequent TA cloning step. The reamplification products were separated on 2% agarose/EtBr gel with 100 bp DNA markers. The band of interest was cut with sterile scalpel blade under UV light and purified using NucleoSpin Extract II Kit (MN, Germany).

### Cloning and Sequencing of cDNA AFLP Fragments

The gel-purified reamplification products were cloned by the TA cloning method using pGEM-T Easy Vector Systems (Promega, US) and freshly prepared DH5α competent cells. The recombinant plasmids were purified with QIAGEN Plasmid Mini Kit (QIAGEN, Germany). Sequencing was performed with BigDye Terminator v3.1 Cycle Sequencing Kit (Applied Biosystems, US) using T7 promoter primer on Applied Biosystems 3130×l Genetic Analyzers.

### *In silico* Sequence Analysis

The sequences free of vector and adapter contamination were searched for homology using the BLASTN and BLASTX algorithm [25] against the public databases in NCBI (the National Centre for Biotechnology Information) and TAIR (The *Arabidopsis* Information Resources). The sequences were annotated based on the Gene Ontology (GO) terms [26] and InterPro (Integrated documentation resource for protein families) terms [27] associated with the respective best BLAST hit. Analysed sequence data was submitted to dbEST division of GenBank, NCBI.

### Time Course Quantitative Real Time RT PCR Analysis

Total RNA was freshly isolated from un-infested as well as 6, 12, 24 and 48 hour time interval aphid-infested samples using TRIZOL Reagent (Invitrogen, US). 1^st^ strand cDNA synthesis was carried out from 2 µg total RNA using iScript cDNA Synthesis Kit for RT PCR (Bio-Rad). The quality was verified based on OD_260_/OD_280_ values and concentration was measured based on OD_260_ values using a ND-1000 Spectrophotometer (Nanodrop, Wilmington, DE, US). Specific real time PCR primers (Table S3) were designed for 25 cDNA AFLP identified differentially induced genes with NCBI/Primer-Blast software.

The 2^nd^ step of the RT PCR was performed with 25 cDNA AFLP fragment specific primers and Taq DNA Polymerase (Fermentas, Thermo Fisher Scientific, US). The RT PCR steps were as follows, initial denaturation for 2 min at 94°C, followed by 35 cycles consisting of denaturation for 30 sec at 94°C, annealing for 30 sec at 55°C and extension for 30 sec at 72°C and final extension for 8 min at 72°C. GADPH gene (NM_111283) was used as the internal control. The RT PCR products were run on 2% agarose/EtBr gels with 100 bp DNA Ladder (Genei, India) to check the specificity of the primers.

Quantitative real time RT PCR reaction was carried out with iQ SYBR Green Supermix (Bio-Rad Laboratories) on a iQ5 Multicolor Real Time PCR Detection System (Bio-Rad laboratories) with 250 ng RT PCR verified cDNA AFLP fragment specific primers (Table S3) specifically designed for real Time PCR and 25 ng 1st strand cDNA. The real time amplification pattern was studied over the 48 hour time course with aphid-infested samples at 6, 12, 24 and 48 hour post infestation. Un-infested sample was the experimental control. The Real time RT PCR profile was as follows, initial denaturation at 95°C for 3 min, followed by 50 cycles for 10 sec at 95°C and 30 sec at 55°C. GADPH was used as the internal control and its expression level was constant in the real time RT PCR analysis. Quantification was based on Ct (Cycle threshold) values and PCR efficiency determined by iQ5 Optical System Software, version 2.0 (Bio-rad Laboratories). The reactions were studied for each gene with three biological replicates. The relative fold value changes with respect to the experimental control at different time points for each gene normalized with internal control were calculated using the 2^−ΔΔCt^ method [28]. One way ANOVA followed by Tukey’s multiple comparison test was carried out to determine the significance of the up or down regulation status of the genes over the time course. Relative gene expression level was considered significant at p<0.05 level.

## Results and Discussion

### Forced Infestation on the Wild Crucifer

**Figure 1:**
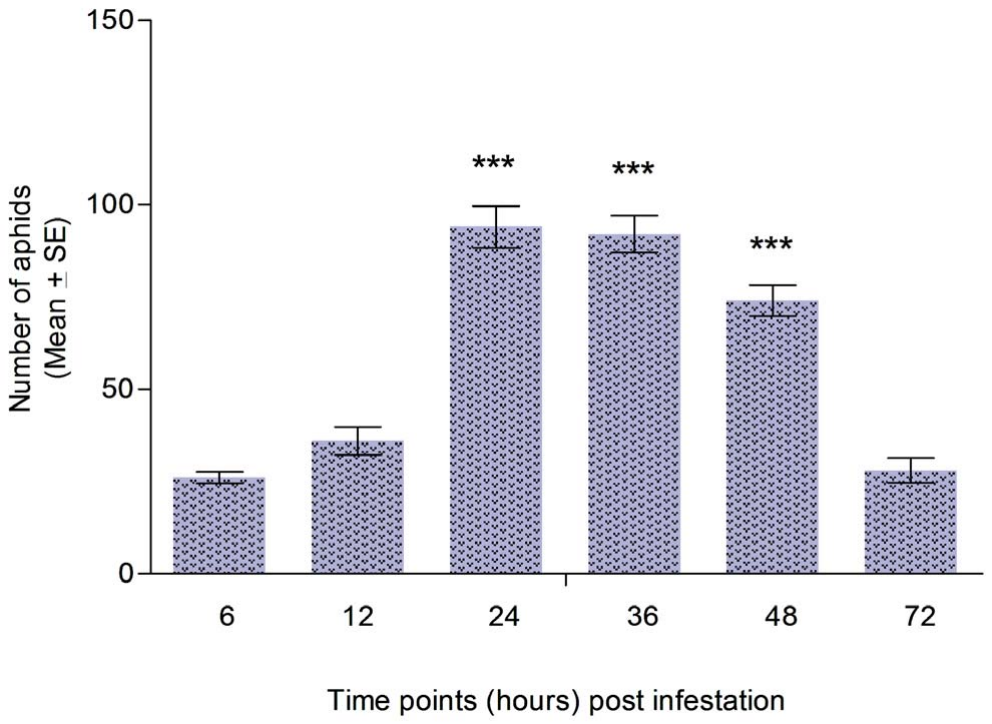
Time course aphid infestation study. Record of the mean number of aphids at different time points post infestation with *L. erysimi* on *R. indica*. Aphid colonization peaks at around 24 hpi and falls sharply by 48 to 72 hpi. Error bars represent standard error of the mean (n = 5). ‘*’, ‘**’ and ‘***’ indicate the level of significance of the noted number of aphids at significance level of p<0.05.

The time course aphid infestation study (Fig. 1) was carried out to precisely determine the experimental time frame for the transcriptomic analysis. Within two hours post infestation most of the aphids, comprising of a mixed population of aptera and nymph, took shelter around the stem and a few under the leaves. A surge in population of nymph and aptera was noted within 24 hours and that persisted up to 36 hours. After 24 hours there was no reproduction. By 48 hours and onwards, the population level started to decline sharply with the appearance of the winged form (alate) of the aphids. By 72 hours, the plants were left only with the nymphs. This 72 hour aphid treatment did not develop any visible disease symptom on the plant and the challenged plants showed normal development like the untreated control plants. Aphid attack, however, neither produced any visible symptom nor retarded plant development at all. Whether this is an example of antixenosis or antibiosis requires separate analysis.

### cDNA AFLP Analysis

**Figure 2:**
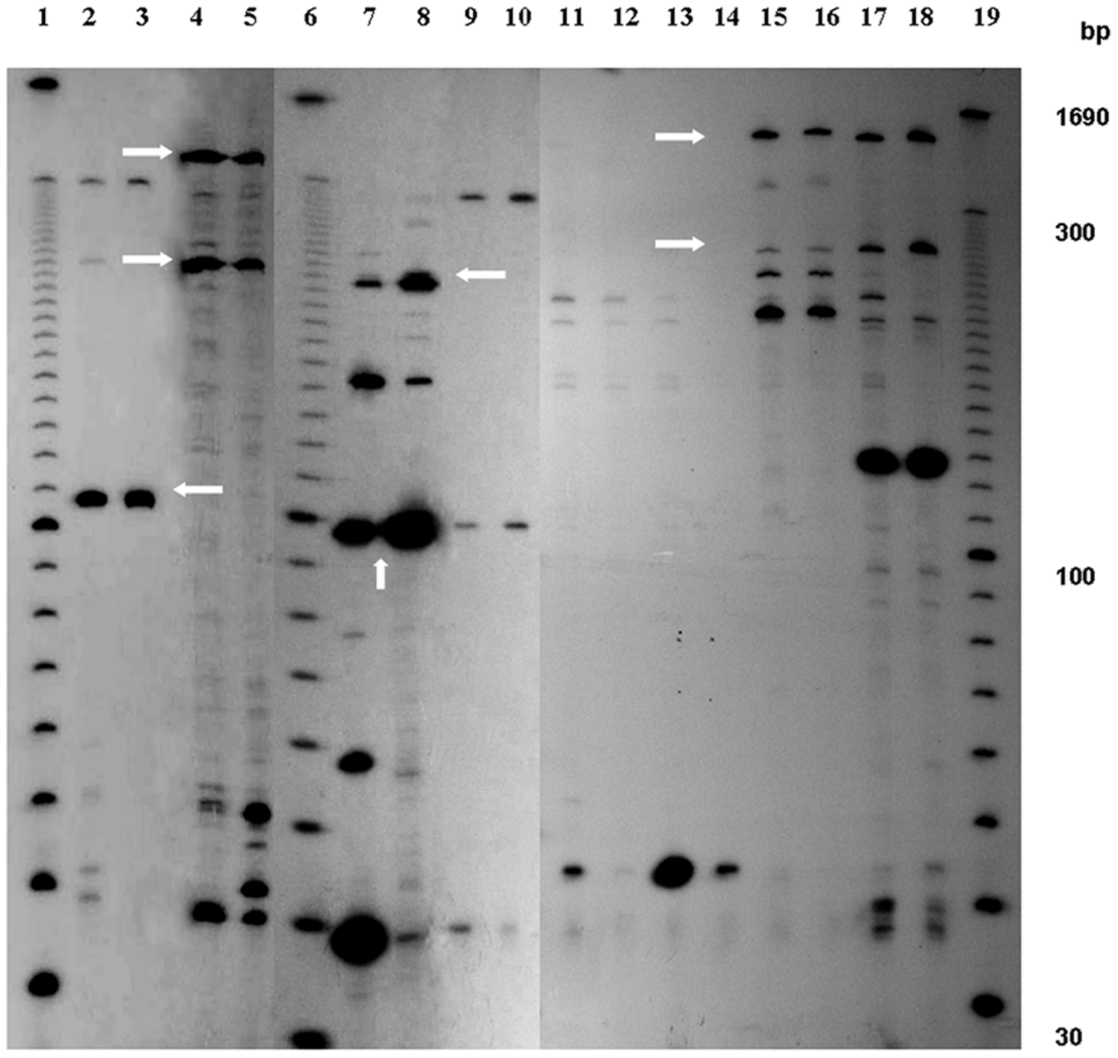
Representative cDNA AFLP Differential Gene Expression Profiles. Between *R. indica* infested with *L. erysimi* (Lanes: 2, 3, 7, 8, 11, 12, 13, 14) and uninfested *R. indica* (Lanes: 4, 5, 9, 10, 15, 16, 17, 18) samples using different primer combinations. Lanes 2, 3, 4, 5 with E-AAC/M-CAT; Lanes 7, 8, 9, 10 with E-AAC/M-CAG; Lanes 11, 12, 13, 14, 15, 16, 17, 18 with E-AAC/M-CTG. Differential bands are indicated by arrows. Lanes 1, 6 and 19 are for 30–330 bp marker AFLP DNA ladder. It is a 6% denaturing polyacrylamide gel.

cDNA AFLP is a reproducible transcriptome analysis technique that can serve as a novel gene identifier irrespective of previous sequence information allowing independent research specially on non-model systems like *R. indica*. In this study the differential gene expression profile (Fig. 2) of the early-to-late defense response was captured by cDNA AFLP analysis between aphid infested and uninfested *R. indica* plants with pooled cDNA samples from 12 to 72 hpi. Whole aerial part of the plants was considered during sampling, as aphid infestation ranged both leaf and stem with dense colonization around the stem. The study was carried out with two biological and two technical replicates. Out of the 40 combinations of EcoR1 and Mse1 specific selective primers (Table S1 & S2) used, 19 combinations produced differential cDNA AFLP profile. The cDNA AFLP fragments ranged from 80 bp to 1000 bp as marked by the 30 to 300 bp AFLP DNA ladder. One hundred and sixteen differentially expressed cDNA AFLP fragments were attempted to clone and sequence.

### *In silico* Sequence Analysis

Selected 116 differentially expressed fragments were eluted from the cDNA AFLP gels and were reamplified for further analysis. Sixty six fragments could be cloned and sequenced successfully. After trimming off the vector and adapter sequences, these 66 sequences were blasted for homology using the BLASTN and BLASTX algorithm [25] against nucleotide and protein sequences respectively in the non-redundant (nr) databases of NCBI and TAIR. The best blast hit was determined from among the score and E-values of the sequences producing significant alignments. The sequences were annotated based on the GO [26] and InterPro terms [27] associated with the respective best BLAST hit. The *in silico* sequence analysis revealed expression of TDFs in the functional categories of signal transduction (31%), photosynthesis (21%), oxidative stress response (13%), wound response (3%), defense response (3%), structure (13%), metabolism (3%) as well as proteins of unknown function (13%). Out of 66 sequences blasted, 30 were unique. Rest 36 sequences were repetitive and were excluded from the list. These 30 expressed sequence tag (EST) sequences (Table 1) were submitted to the dbEST division of GenBank, NCBI for accession (Accession Numbers: JK034053, JK034054, JK034055, JK034056, JK034057, JK034058, JK034059, JK034060, JK034061, JK034062, JK034063, JK034064, JK034082, JK034065, JK034066, JK034067, JK034068, JK034069, JK034070, JK034071, JK034072, JK034073, JK034074, JK034075, JK034076, JK034077, JK034078, JK034079, JK034080, JK034081).

**Table 1:** The candidate resistance response TDFs identified in the transcriptomic analysis of *R. indica- L. erysimi* interaction.

ESTNo.	AccessionNo.	Size(bp)	E-Value	cDNA AFLPProfile	Annotation	Gen BankHomolog	Functionalcategory
RI01	JK034053	76	3e-31	Induced	Cytosol leucyl aminopeptidase family protein	AT2G2400	Wound response
RI02	JK034054	407	5e-71	Induced	Plant defensin 1.2c	AT5G44430	Defense response
RI03	JK034055	355	2e-45	Repressed	Class I glutamine amidotransferase likesuperfamily protein	AT3G54600	Metabolic
RI04	JK034056	240	2e-44	Repressed	Enhanced downey mildew 2; EDM2	AT5G55390	Signalling
RI05	JK034057	440	8e-70	Repressed	Auxine response factor 19, ARF19	AT1G19220	Signalling
RI06	JK034058	383	e-105	Induced	PDX1.3, Aldolase type TIM barrel family protein	AT5G01410	Photosynthesis
RI07	JK034059	362	3e-75	Repressed	TCP family transcription factor 4	AT3G15030	Signalling
RI08	JK034060	148	5e-44	Repressed	Remorin family protein	AT2G45820	Signalling
RI09	JK034061	285	4e-52	Repressed	S-adenosyl-L-methionine dependent methyltransferases	AT1G78140	Signalling
RI10	JK034062	626	0.0	Induced	Serine transhydroxymethyltransferase 1	AT4G37930	Oxidative stress
RI11	JK034063	333	e-115	Repressed	ENT plant tudor like domains containing protein	AT3G12140	Signalling
RI12	JK034064	507	e-133	Repressed	Glutaredoxin family protein	AT5G13810	Oxidative stress
RI13	JK034082	203	2e-38	Repressed	Small nuclear ribonucleoprotein family protein	AT2G18740	Structural
RI14	JK034065	89	5e-27	Repressed	HSPRO2, Ortholog of sugar beet, HS1 ^PRO-1^	AT2G40000	Signalling
RI15	JK034066	223	1e-48	Induced	Ribulose bisphosphate carboxylase small chain 1A	AT1G67090	Photosynthesis
RI16	JK034067	364	e-135	Repressed	HEMB1, Aldolase superfamily protein	AT1G69740	Photosynthesis
RI17	JK03406	296	1e-98	Induced	Photosystem I subunit L; PSAL	AT4G12800	Photosynthesis
RI18	JK034069	74	4e-30	Repressed	Glutathione S-Transferase TAU 20	AT1G78370	Oxidative stress
RI19	JK034070	186	4e-54	Repressed	Ribosomal protein L35Ae family protein	AT1G74270	Structural
RI20	JK034071	177	8e-37	Repressed	Unknown protein	AT3G46220	Unknown
RI21	JK034072	212	2e-28	Repressed	Unknown protein	AT5G14110	Unknown
RI22	JK034073	305	e-112	Repressed	Glycoprotein membrane precursor GPI anchored	AT3G06035	Signalling
RI23	JK034074	69	5e-17	Repressed	Pollen Ole e1 allergen and extension family protein	AT5G22430	Unknown
RI24	JK034075	138	4e-41	Repressed	Unknown protein	AT4G14830	Unknown
RI25	JK034076	298	3e-93	Repressed	Tic 22 like family protein	AT5G62650	Structural
RI26	JK034077	716	e-107	Repressed	Coatomer beta subunit	AT4G31480	Structural
RI27	JK034078	165	2e-31	Induced	PS II oxygen evolving complex 1, PSBO-1	AT5G66570	Photosynthesis
RI28	JK034079	123	8e-33	Induced	RBCSIA	AT1G67090	Photosynthesis
RI29	JK034080	190	8e-65	Repressed	FKBP like peptidyl prolyl cis trans isomerasefamily protein	AT5G13410	Signalling
RI30	JK034081	500	2e-77	Induced	Thioredoxin superfamily protein, ATPRXQ	AT3G26060	Oxidative stress

### Time Course Relative Gene Expression Analysis

**Figure 3:**
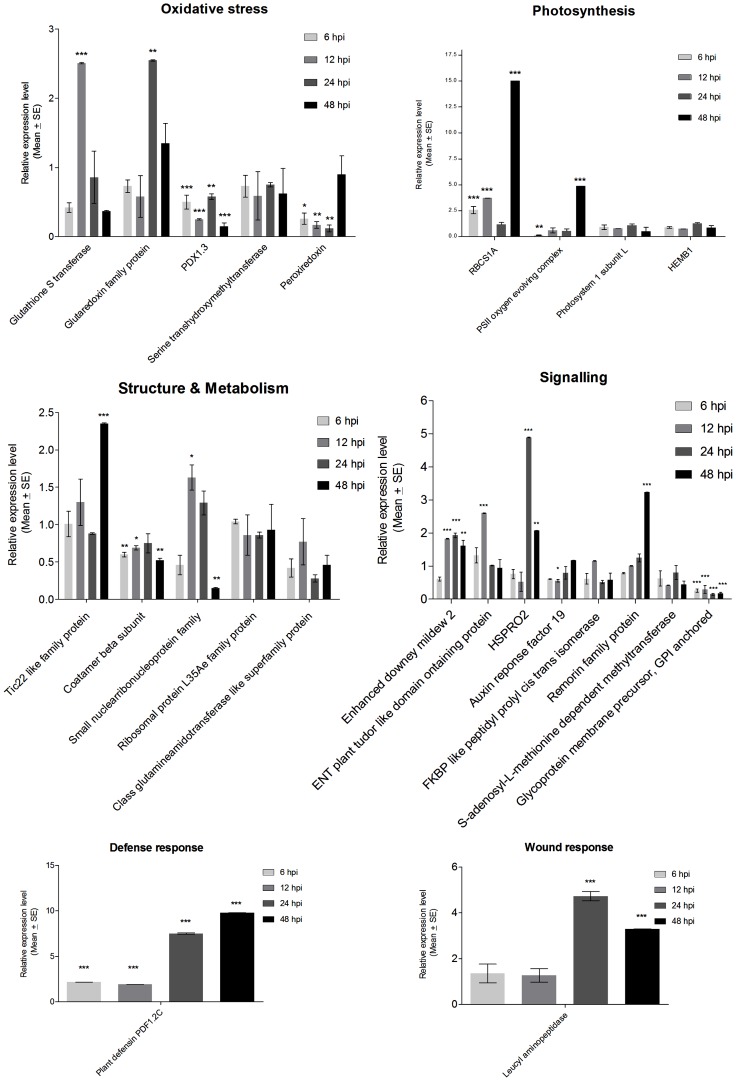
Time course relative gene expression analysis. Time course relative expression (Y-axis) level profiles of 24 genes induced in *R. indica* on forced infestation with *L. erysimi* (as identified by cDNA AFLP with respect to uninfested plants) at different time points viz. 6, 12, 24, 48 hours post infestation (X-axis), as analysed quantitative real time RT PCR analysis. The genes have been represented under different functional categories. Error bars represent standard error of the mean (n = 3). ‘*’, ‘**’ and ‘***’ indicate the level of significance of the relative gene expression level noted at the significance level of p<0.05.

The cDNA AFLP analysis was attempted to note the differential gene expression profile in the wild plant on aphid infestation. However, since the study was done on pooled RNAs from different time points (12 hpi to 72 hpi), we could register only the differentially expressed genes as either induced or repressed (Table 1). A detailed time course relative gene expression analysis was pursued with the gene specific primers for 25 cDNA AFLP fragments (excluding the fragments with unknown function) by quantitative real time RT PCR to cover the early-to-late aphid induced defense response (at 6, 12, 24 and 48 hpi) and understand the temporal defense signalling pattern in *R. indica* against mustard aphid *L. erysimi.* Primers for TCP family transcription factor 4 failed to show any amplification in real time RT PCR analysis. Hence 24 cDNA AFLP fragments could be analysed eventually (Fig. 3; Table S4). The temporal real time RT PCR analysis let us get a much better understanding of the differential gene expression status of the cDNA AFLP identified fragments over post infestation time course. Out of 24 fragments, 17 fragments showed significant up or down regulation at significance level of p<0.05 (Fig. 3). The difference in gene expression regulation noted by the two separate methods could have arisen due to different sampling and analysis pattern. The cDNA AFLP profiling was carried out with pooled RNAs from different time points (12 hpi, 24 hpi, 48 hpi and 72 hpi). The real time RT PCR study was carried out with separate RNAs for every time point analysed (6 hpi, 12 hpi, 24 hpi and 48 hpi) for each gene. Thus it was not possible as such to compare the observed difference in expression profiles by these two methods. However, we have noted the expression status of each gene identified and analysed by both methods in Table 1 (cDNA AFLP profile) and Table S4 (real time RT PCR profile).

### Transcriptional Reprogramming in Response to Aphid Feeding

#### Oxidative stress response

The disturbance in membrane potential, cytosolic [Ca^2+^] and local as well as systemic ROS accumulation by wound and aphid salivary components are the earliest molecular messengers in plant aphid interaction leading to redox imbalance [29]. The foremost cellular concern is thus manifested by up regulation of potential redox regulators. Expressions of redox regulators however are not uniformly regulated on induction by aphid feeding [30]. TDF for SA induced redox regulator glutathione S-transferase (AT1G78370) was up regulated to 2.51 fold early at 12 hpi but showed down regulation in the following time points. TDF for glutaredoxin family protein (AT5G13810) showed up regulation at 24 hpi up to 2.55 fold and came down to 1.35 fold at 48 hpi. Down regulation was noted for antioxidant enzyme peroxiredoxin, ATPRX Q, a thioredoxin superfamily protein (AT4G37930) throughout the time course. The *de novo* vitamin B6 biosynthetic genes PDX1 and PDX2 in *A. thaliana* are up regulated in response to abiotic stress and function as antioxidant in oxidative stress. Mutants deficient in PDX1.3 have been noted to be sensitive to osmotic, UV and singlet oxygen stress [31], [32]. However, TDFs for pyridoxine biosynthetic enzyme PDX1.3 (AT5G01410) was noted to be down regulated throughout in the present study. There are some reported instances of association of photorespiration with disease resistance against biotrophic and necrotrophic pathogens [33], [34], [35]. In this study, down regulation was noted for photorespiratory enzyme serine transhydroxymethyltransferase, SHMT1 (AT4G37930).

#### Signalling signature

Each type of host predator interaction is unique and is best identified by the respective induced signalling signature. Signal transduction category was the most prominent category showing differential regulation of gene expression in *R. indica*-*L. erysimi* interaction. RPP7 is a NBS-LRR type disease resistance protein that confers specific disease resistance against downey mildew causing oomycetes *Hyaloperonospora parasitica* isolate *Hiks1* (*HpHiks1*) in *Arabidopsis thaliana*. The present study reports the up regulation of TDF for EDM2 (AT5G55390), the transcriptional regulator for RPP7, up to 1.93 fold by 24 hpi and 1.61 fold at 48 hpi. EDM2 operates earlier upstream of defense associated SA dependent reactive oxygen species production as well as hypersensitive response [36]. The simultaneous up regulation (2.6 fold at 12 hpi) of TDF for EMSY N-terminal/plant Tudor like domain containing protein (AT3G12140) reported also to act in RPP7 immunity in *R. indica*-*L. erysimi* interaction, makes the involvement of RPP7 in *L. erysimi* defense signalling more promising [37]. Similar identification in this category, TDF for HSPRO2 (AT2G40000) is an LRR-containing protein from *Arabidopsis* with homology to Hs1^PRO-1^, which confers resistance to the beet cyst nematode (*Heterodera schachtii* Schmidt). HSPRO2 expression is induced by SA and repressed by JA/ET [38]. HSPRO2 level was up regulated to 4.89 fold by 24 hpi and 2.07 fold by 48 hpi. Members of the auxin signalling network including the ARF genes are involved in abiotic and biotic stress responses besides growth and developmental processes. Auxin response is enhanced by JA/ET but repressed by SA [39]. TDF for ARF 19 (AT1G19220) identified in this reaction revealed basal level expression in the real time RT PCR study. Peptidyl-prolyl cis-trans isomerase activity is important for specific aspects of auxin regulation of plant growth, development, and environmental responses [40]. TDF identified for FKBP like peptidylprolyl cis-trans isomerase family protein (AT5G13140) identified in this study also displayed basal level expression besides ARF 19 suggesting possibly the unaffected development of the aggressive weed even under herbivore pressure. The plant specific protein remorin is a hydrophilic oligogalacturonide (OGA) binding protein that interacts with receptor like kinases in regulating bacterial infection as a scaffold protein and in virus macromolecular trafficking as functional lipid rafts [41], [42]. A 3.23 fold up regulation at 48 hpi was noted for the TDF for remorin family protein (AT2G45820). Highly reactive S-adenosyl-L-methionine often used as the methyl donor by the methyl transferases. TDF for S-adenosyl methyltransferase (AT1G78140) was noted to express at basal level in this study. Glycosylphosphatidylinositol (GPI) anchored membrane proteins participate in cell wall remodelling, defense responses, and cell signalling [43]. TDF for GPI anchored glycoprotein membrane precursor (AT3G06035) identified in this study was down regulated throughout the analysis. TDF for the TCP family transcription factor 4 (AT3G15030) required for the JA biosynthetic enzyme lipoxygenase2 (LOX2) expression in development but not in wound response was noted to be down regulated in the cDNA AFLP analysis [44]. However, the time course quantitation of the expression could not be carried out as the primers of TCP family transcription factor 4 failed to show any amplification in the real time RT PCR analysis.

#### Civilian service

The direct and indirect damage of plant tissues caused by wound and redox-imbalance leads to the reprogramming of housekeeping genes in order to maintain cellular homeostasis. Thus aphid feeding leads to nutrient depletion. The deficiency can be overcome by increasing photosynthesis and metabolic reprogramming. However, this seems costly when defense is up in the priority list. Thus some studies have reported decrease in photosynthetic rates on aphid attack [45], [46]. In the present study of resistant response in a wild aggressive plant, ribulose bisphosphate carboxylase small chain 1A (AT1G67090) showed up regulation early at 6 and 12 hpi, was down regulated at 24 hpi and culminated at 48 hpi to 15 fold. PSII oxygen evolving complex 1 (AT5G66570) was initially down regulated up to 24 hpi and showed 4.86 fold up regulation at 48 hpi. TDF for 5-aminolevulinate dehydratase, HEMB1 (AT1G69740) and photosystem 1 subunit L, PSAL, (AT4G12800) however, revealed unaffected expression in the real time RT PCR analysis. The only differentially expressed metabolic candidate Class 1 glutamine amidotransferase (AT3G54600) showed down regulation in the time course study. Among the up regulated structural TDFs were small nuclear ribonucleoprotein (AT2G18740) (up regulated to 1.63 fold at 12 hpi) and chloroplast inner envelope Tic22 like family protein (AT5G62650) (up regulated to 2.35 fold at 48 hpi) involved in import of nuclear encoded proteins from cytoplasm [47]. TDFs for COP-1 vesicle coat, coatomer beta subunit (AT4G31480) involved in Golgi trafficking in the retrograde sorting pathway and ribosomal protein L35Ae (AT1G74270) was noted to have basal level expression throughout the real time RT PCR analysis.

#### Enzyme inhibitors

Sap suckers like aphids were long supposed to lack digestive enzymes [48]. However, recent studies reveal putative protease activity in aphids like *Myzus persicae* [49], [50] and *Aphis gossypii* [51]. These revelations suggest presence of corresponding enzyme inhibitors in the plants as plant-insect defense reaction is the result of an evolutionary arms race. Thus around 28 leucyl amino peptidases [52] and thirteen putative defensin genes [53] have been noted in the *Arabidopsis* genome. This study reports 4.73 fold up regulation at 48 hpi of wound responsive cytosol leucyl aminopeptidase (AT2G2400) that works with arginase in the Lepidopteran midgut to disturb midgut integrity and thereby decreases the availability of essential amino acid arginine [54]. Its expression is induced by JA and systemin and suppressed by SA [52]. Plant defensins are small basic, cystein rich peptides. They are expressed at high levels constitutively in seeds and roots to provide the first line of defense against soil borne pathogens [53]. In this study TDF for plant defensin family protein PDF1.2C showed up regulation from early 6 hpi and peaked at 48 hpi to 9.78 fold. PDF1.2C induction requires JA and ET concomitantly but is independent of SA [55].

#### Unknown function

In addition to the above listed candidates, TDFs for three hypothetical proteins (AT3G46220, AT5G14110, AT4G14830) and Ole e 1 allergen and extension family protein (AT5G22430) of unknown function were also noted to be expressed.

## Conclusion

We were inspired by the successful introgression of the aphid resistance trait from the wild crucifer *R. indica* to the susceptible *B. juncea* by somatic hybridization, as demonstrated by the somatic hybrids and their backcross-progenies [20], [21]. In order to decipher the wild secrets, a molecular insight was necessary and thus a transcriptomic exploration of the resistant response was made by forced aphid infestation compared to the un-infested plants. The forced infestation study observed mustard aphid feeding and reproduction unobstructed for 24 to 36 hours on the non-host *R. indica*. After 24 hpi no aphid reproduction was noted. Emergence of the winged morphs (alates) started after 24 hpi suggesting defense response at the plant end leading to host rejection by the aphid. This was supported by the transcriptomic data as well. Among the transcriptomic identifications signalling candidates comprised the largest group suggesting a prominent aphid induced defense signalling. Phytohormonal cross talk plays an important role in plant defense signaling. The general view of plant disease resistance is that SA dependent signalling is effective against biotrophs and JA/ET dependent signalling against necrotrophs [56]. The compatible plant aphid interaction is often successful due to the decoy defense strategy played by the aphids by inducing the less effective SA dependent defense response at the cost of suppressing the more effective JA dependent pathways [30], [45], [57], [58], [59], [60], [61]. The present incompatible interaction study revealed differential regulation of SA (glutathione S-transferase, glutaredoxin, HSPRO2), JA/ET (PDF1.2c) and JA/Systemin (leucyl aminopeptidase) responsive genes. Glutaredoxin GRX480 is considered as one of the regulators in SA/JA cross talk. GRX480 expression is inducible by SA inducible NPR1. In collaboration with TGA transcription factors, GRX480 has been noted to suppress a subset of the JA responsive genes including PDF1.2; however, has no effect on LOX2 or VSP2 [62], [63]. While the SA responsive genes including glutathione S-transferase, glutaredoxin and HSPRO2 showed a downhill temporal expression pattern in the present study, JA signature gene PDF1.2c (up regulated throughout the study) showed a gradual temporal increase in the expression level noted up to latest 48 hpi. This observation tempts to suggest a possible JA/SA cross talk leading to JA dominance in the later phase cancelling the promise of decoy trap leading to effective resistance. However, no conclusion should be drawn at this point regarding hormonal signaling behind aphid resistance. Further molecular and functional characterization of individual identifications (including the proteins with unknown function) and additional candidates is necessary as well to get a better grasp about the wild aphid resistance mechanism. The induced R genes for viz. LRR protein HSPRO2 and NBS-LRR protein RPP7 (as suggested by the up regulation of its transcriptional regulator EDM2 and EMSY N-terminal/plant Tudor like domain containing protein in the study) are likely the direct candidates to check for, in developing mustard aphid resistance in *Brassica*. Thus full length sequencing and further characterization of these two genes from *R. indica* have been initiated as a continuation of this work.

Search for control over mustard aphid is a long battle in the tropical agro-research. We are presenting here the first molecular data for the *R. indica-L.erysimi* incompatible interaction. We are hopeful that our result will contribute in understanding the resistance signalling signature of the aggressive wild crucifer *R. indica* and mimicking the same in consistently susceptible *Brassica*s to successfully defend the irresistible aphid attack in the field.

## Supporting Information

Table S1 Primer sequences of cDNA AFLP primers.
(DOCX)

Table S2 Primer combinations used in cDNA AFLP analysis.
(DOCX)

Table S3 Real time RT PCR primers.
(DOCX)

Table S4 Relative expression levels of the cDNA AFLP identified defense response candidates.
(DOCX)
