# Supplementary material for: Identification of Genes Involved in Wild Crucifer Rorippa indica Resistance Response on Mustard Aphid Lipaphis erysimi Challenge
Source: PLoS One. 2013 Sep 9;8(9):e73632. doi: 10.1371/journal.pone.0073632 (PMC3767759; doi:10.1371/journal.pone.0073632)
Supplement: Table S1 — Primer sequences of cDNA AFLP primers. (DOCX) [file pone.0073632.s001.docx]

**Table S1** Primer sequences of cDNA AFLP primers

| **Primer** | **Sequence (5´-3´)** |
| --- | --- |
| E-AAC | GACTGCGTACCAATTC-AAC |
| E-AAG | GACTGCGTACCAATTC-AAG |
| E-ACA | GACTGCGTACCAATTC-ACA |
| E-ACC | GACTGCGTACCAATTC-ACC |
| E-ACG | GACTGCGTACCAATTC-ACG |
| E-ACT | GACTGCGTACCAATTC-ACT |
| E-AGC | GACTGCGTACCAATTC-AGC |
| E-AGG | GACTGCGTACCAATTC-AGG |
| M-CAA | GATGAGTCCTGAGTAA-CAA |
| M-CAC | GATGAGTCCTGAGTAA-CAC |
| M-CAG | GATGAGTCCTGAGTAA-CAG |
| M-CAT | GATGAGTCCTGAGTAA-CAT |
| M-CTA | GATGAGTCCTGAGTAA-CTA |
| M-CTC | GATGAGTCCTGAGTAA-CTC |
| M-CTG | GATGAGTCCTGAGTAA-CTG |
| M-CTT | GATGAGTCCTGAGTAA-CTT |
