# Supplementary material for: Identification of Genes Involved in Wild Crucifer Rorippa indica Resistance Response on Mustard Aphid Lipaphis erysimi Challenge
Source: PLoS One. 2013 Sep 9;8(9):e73632. doi: 10.1371/journal.pone.0073632 (PMC3767759; doi:10.1371/journal.pone.0073632)
Supplement: Table S2 — Primer combinations used in cDNA AFLP analysis. (DOCX) [file pone.0073632.s002.docx]

**Table S2** Primer combinations used in cDNA AFLP analysis

| **Combination No.** | **Primer Combination** | **Combination No.** | **Primer Combination** |
| --- | --- | --- | --- |
| 1. | E-AAC/ M-CAA | 21. | E-ACA/M-CTA |
| 2. | E-AAC/M-CAC | 22. | E-ACA/M-CTC |
| 3. | E-AAC/M-CAG | 23. | E-ACA/M-CTG |
| 4. | E-AAC/M-CAT | 24. | E-ACA/M-CTT |
| 5. | E-AAC/M-CTA | 25. | E-ACC/M-CAA |
| 6. | E-AAC/M-CTC | 26. | E-ACC/M-CAC |
| 7. | E-AAC/M-CTG | 27. | E-ACC/M-CAG |
| 8. | E-AAC/M-CTT | 28. | E-ACC/M-CAT |
| 9. | E-AAG/M-CAA | 29. | E-ACC/M-CTA |
| 10. | E-AAG/M-CAC | 30. | E-ACC/M-CTC |
| 11. | E-AAG/M-CAG | 31. | E-ACC/M-CTG |
| 12. | E-AAG/M-CAT | 32. | E-ACC/M-CTT |
| 13. | E-AAG/M-CTA | 33. | E-ACG/M-CAA |
| 14. | E-AAG/M-CTC | 34. | E-ACG/M-CAC |
| 15. | E-AAG/M-CTG | 35. | E-ACG/M-CAG |
| 16. | E-AAG/M-CTT | 36. | E-ACG/M-CAT |
| 17. | E-ACA/M-CAA | 37. | E-ACG/M-CTA |
| 18. | E-ACA/M-CAC | 38. | E-ACG/M-CTC |
| 19. | E-ACA/M-CAG | 39. | E-ACG/M-CTG |
| 20. | E-ACA/M-CAT | 40. | E-ACG/M-CTT |
