# Supplementary material for: Identification of Genes Involved in Wild Crucifer Rorippa indica Resistance Response on Mustard Aphid Lipaphis erysimi Challenge
Source: PLoS One. 2013 Sep 9;8(9):e73632. doi: 10.1371/journal.pone.0073632 (PMC3767759; doi:10.1371/journal.pone.0073632)
Supplement: Table S3 — Real time RT PCR primers. (DOCX) [file pone.0073632.s003.docx]

Table S3: Real time RT PCR primers

| **GenBank Accession No.** | **Gene annotation** | **Sequences (5´-3´)** | **GenBank Accession No** | **Gene annotation** | **Sequences (5´-3´)** |
| --- | --- | --- | --- | --- | --- |
| JK034053 | Cytosol leucyl aminopeptidase | Forward: AACAAGCGTAGCAAC | JK034065 | HSPRO2, ortholog of sugar | Forward: ACGGTGGTCGATGTACGG |
|  | family protein | Reverse: ATAGACATGGCTGGACCGGT |  | beet HS1^PRO-1^ | Reverse: CCGGTTCTCCAAGCTTTGGA |
| JK034054 | Plant defensin 1.2c | Forward: ATCAACCTTGAGGGAGCACG | JK034066 | Ribulose bisphosphate carboxylase | Forward: GTTAGCCTTACGGGTCACCG |
|  |  | Reverse: ATACGCAAACTTAGCACCAAAGC |  | small chain 1A; RBCS1A | Reverse: CAACGCAGAGTACATGGGGG |
| JK034055 | Class I glutamine amidotransferase | Forward: TGGCATGTAAAGTGGACGCA | JK034067 | HEMB1. Aldolase superfamily | Forward: ACGTCGTAACCGTGCATCTC |
|  | like superfamily protein | Reverse: CCGTCCTCGAGGTCATAAACG |  | protein | Reverse: GCAACCAGGCATAGCTCCAA |
| JK034056 | Enhanced downey mildew 2 | Forward: CGGAGCGTCAAGGAGAAAGTT | JK034068 | Photosystem I subunit L; PSAL | Forward: CGGAACGGTGAAGGAAGAGAC |
|  |  | Reverse: CCTCAAAGCTTTTCCTCGGATTG |  |  | Reverse: TCTTCTGCTTCTCTTTCGAGGC |
| JK034057 | Auxine response factor 19 | Forward: TCCTCCAGTAAGCTCGTCCTT | JK034069 | Glutathione s-transferase TAU20 | Forward: GATATGGGGGAAGAAAGGTGAGG |
|  |  | Reverse: AAGGGCTGTTGATTCTGAGACG |  |  | Reverse: ACAAGAATCTTCACTGC |
| JK034058 | PDX1.3, Aldolase type TIM barrel | Forward: AGCATCTGAGCCAAACCGAC | JK034070 | Ribosomal protein L35Ae | Forward: AAATGGTGAAGGGACGCCAA |
|  | family protein | Reverse: ATAATGGAAGGAACCGGCGTC |  | family protein | Reverse: TCCTCTTGGGTGTTGACTCCT |
| JK034059 | TCP family transcription factor 4 | Forward: GCATTCGTTCCCGGATGGT | JK034073 | Glycoprotein membrane | Forward: GAACAAGCCTTGCACCAACG |
|  |  | Reverse: ACTGCGTACCAATTCACCGAG |  | precursor GPI anchored | Reverse: GTGAGGACGAGGTTTGGGTC |
| JK034060 | Remorin family protein | Forward: GCAGAAGAGAAGAGAGCAATGGT | JK034076 | Tic 22 like family protein | Forward: TCTCTCTTCGATCTGTACCGGG |
|  |  | Reverse: GCTTTTGGAACGATGCCAGTG |  |  | Reverse: CAGGAGAGGCTGCACAGATG |
| JK034061 | S-adenosyl L-methionine dependent | Forward: GTCCCTTCTGTTATAGCTCCTTAGC | JK034077 | Coatomer beta subunit | Forward: GCCAAAATCGCGTTCCTACG |
|  | methyltransferases superfamily protein | Reverse: GAGGCATTGGTTCACTGTATTGC |  |  | Reverse: TGCGGTTCCTTAGCCGATTG |
| JK034062 | Serine transhydroxymethyl | Forward: TCAGGGAAACAAGCTGAAGGAC | JK034078 | PSII oxygen evolving complex, | Forward: CTTGGCCGACTGGAGGAATG |
|  | transferase 1, SHMT1 | Reverse: TTGGGAACTGCTTAGCGGATT |  | PSBO-1 | Reverse: CAACGCAGAGTACATGGGGG |
| JK034063 | EMSY N terminus (ENT) , plant tudor | Forward: ACTTGGACCACCACCTACACA | JK034080 | FKBP like peptidyl prolyl cis | Forward: CGATTAGGAACTGGGCCTACAC |
|  | like domains containing protein | Reverse: TGCAATGGCAGCAATAAGCG |  | trans isomerise family protein | Reverse: TCCTGAGTAACCTATTGTGTAACCG |
| JK034064 | Glutaredoxin family protein | Forward: CACGGATATCGCCGTTTACGA | JK034081 | Thioredoxin superfamily | Forward: TGAGATCGAACTCTGCTTCGTG |
|  |  | Reverse: ATCCATGGACGTATCGCGTTC |  | protein, ATPRX Q | Reverse: TGACTGCGTACCAATTCAAGCA |
| JK034082 | Small nuclear ribonucleoprotein | Forward: CAAGGAAGTGGCACAAGTTGC | NM_111283 | Glyceraldehyde 3-phosphate | Forward: ACCACTAACTGCCTTGCTCC |
|  | family protein | Reverse: AATCCGCGACAGAGTTTGGT |  | dehydrogenase | Reverse: AAGCAGCTCTTCCACCTCTC |
