# Supplementary material for: Identification of Genes Involved in Wild Crucifer Rorippa indica Resistance Response on Mustard Aphid Lipaphis erysimi Challenge
Source: PLoS One. 2013 Sep 9;8(9):e73632. doi: 10.1371/journal.pone.0073632 (PMC3767759; doi:10.1371/journal.pone.0073632)
Supplement: Table S4 — Relative expression levels of the cDNA AFLP identified defense response candidates. (DOCX) [file pone.0073632.s004.docx]

Table S4: Relative expression levels of the cDNA AFLP identified defense response candidates

| Defense response candidates | | Relative expression levels (2^-∆∆Ct^) at different time points [hour post infestation (hpi)] | | | |
| --- | --- | --- | --- | --- | --- |
| EST No. | Annotation | 6 hpi | 12 hpi | 24 hpi | 48 hpi |
| RI01 | Cytosol leucyl aminopeptidase family protein; AT2G24200 | 1.36±0.41 | 1.27±0.3 | 4.73±0.2 | 3.29±0.01 |
| RI02 | Plant defensin 1.2c; AT5G44430 | 2.16± 0.01 | 1.91±0.01 | 7.49±0.08 | 9.78±0.01 |
| RI03 | Class I glutamine amidotransferase like superfamily protein; AT3G54600 | 0.42±0.12 | 0.77±0.31 | 0.28±0.05 | 0.46±0.13 |
| RI04 | EDM2; Enhanced Downey Mildew 2; AT5G55390 | 0.61± 0.06 | 1.83±0.00 | 1.93±0.07 | 1.61±0.17 |
| RI05 | Auxin response factor 19, ARF19, IAA22, ARF11; AT1G19220 | 0.61±0.01 | 0.56±0.04 | 0.8±0.19 | 1.17±0.01 |
| RI06 | PDX1.3, ATPDX1 Aldolase-type TIM barrel family protein.; AT5G01410 | 0.50±0.10 | 0.25±0.01 | 0.58±0.04 | 0.15±0.05 |
| RI08 | Remorin family protein; AT2G45820 | 0.79±0.02 | 1.01±0.01 | 1.25±0.12 | 3.23±0.01 |
| RI09 | S-adenosyl L-methionine dependent methyltransferase superfamily protein; AT1G78140 | 0.63±0.23 | 0.42±0.01 | 0.81±0.21 | 0.44±0.11 |
| RI10 | Serine transhydroxymethyltransferase 1; SHM1, STM, SHMT1, AT4G37930 | 0.73±0.16 | 0.59±0.35 | 0.75±0.03 | 0.62±0.37 |
| RI11 | EMSY N terminus (ENT), plant tudor like domain containing protein; AT3G12140 | 1.33±0.23 | 2.60±0.01 | 1.02±0.01 | 0.94±0.26 |
| RI12 | Glutaredoxin family protein; AT5G13810 | 0.73±0.09 | 0.58±0.3 | 2.55±0.01 | 1.35±0.29 |
| RI13 | Small nuclear ribonucleoprotein family protein; AT2G18740 | 0.46±0.13 | 1.63±0.17 | 1.29±0.16 | 0.15±0.01 |
| RI14 | HSPRO2, ATHSPRO2, Ortholog of sugar beet HS1 ^PRO-1^; AT2G40000 | 0.76±0.14 | 0.53±0.29 | 4.89±0.01 | 2.07±0.01 |
| RI15 | Ribulose bisphosphate carboxylase small chain 1A,RBCS1A; AT1G67090 | 2.57±0.34 | 3.71±0.01 | 1.18±0.2 | 15±0.01 |
| RI16 | HEMB1, Aldolase superfamily protein; AT1G69740 | 0.89±0.09 | 0.75 ±0.01 | 1.27±0.1 | 0.85±0.22 |
| RI17 | Photosystem I subunit L,PSAL; AT4G12800 | 0.91±0.22 | 0.78±0.01 | 1.08±0.16 | 0.5±0.4 |
| RI18 | ATGSTU20, GSTU20, Glutathione S-transferase TAU 20, GSTU20*;* AT1G78370 | 0.42±0.07 | 2.51±0.01 | 0.86±0.38 | 0.37±0.01 |
| RI19 | Ribosomal protein L35Ae family protein; AT1G74270 | 1.04±0.03 | 0.86±0.27 | 0.86±0.04 | 0.93±0.34 |
| RI22 | Glycoprotein membrane precursor GPI anchored; AT3G06035 | 0.26±0.05 | 0.3±0.12 | 0.15±0.03 | 0.17±0.04 |
| RI25 | Tic22 like family protein; AT5G62650 | 1.01±0.17 | 1.3±0.31 | 0.88±0.01 | 2.35±0.01 |
| RI26 | Coatamer beta subunit; AT4G31480 | 0.6±0.03 | 0.69±0.03 | 0.75±0.13 | 0.52±0.03 |
| RI27 | PSII oxygen evolving complex; PSBO-1, AT5G66570 | 0.14±0.03 | 0.61±0.23 | 0.55±0.2 | 4.86±0.01 |
| RI29 | FKBP like peptidyl prolyl cis trans isomerase family protein; AT5G13140 | 0.62±0.16 | 1.16±0.01 | 0.52±0.05 | 0.59±0.2 |
| RI30 | Peroxiredoxin, thioredoxin superfamily protein, ATPRX Q; AT3G26060 | 0.26±0.08 | 0.17±0.05 | 0.12±0.05 | 0.9±0.27 |
